# Tetra­kis(triphenyl­phosphane-κ*P*)silver(I) trifluoro­acetate ethanol monosolvate

**DOI:** 10.1107/S1600536812045060

**Published:** 2012-11-28

**Authors:** Seik Weng Ng

**Affiliations:** a Department of Chemistry, University of Malaya, 50603 Kuala Lumpur, Malaysia; b Chemistry Department, King Abdulaziz University, PO Box 80203 Jeddah, Saudi Arabia

## Abstract

In the title solvated salt, [Ag(C_18_H_15_P)_4_](CF_3_CO_2_)·C_2_H_5_OH, the Ag^I^ atom is coordinated by four P atoms from triphenyl­phosphane ligands in a distorted tetra­hedral geometry. The anion and solvent are engaged in weak O—H⋯O hydrogen bonds. Of the four triphenyl­phosphane ligands, two each have an equally disordered phenyl ring while the Ag^I^ atom is disordered over two positions in a 0.9595 (15):0.0405 (15) ratio and the trifluoro­acetate anion is equally disordered over two positions with respect to the lattice ethanol mol­ecule.

## Related literature

For a related compound [Ag(C_18_H_15_P)_2_(CF_3_CO_2_)], see: Ng (1998 ▶).
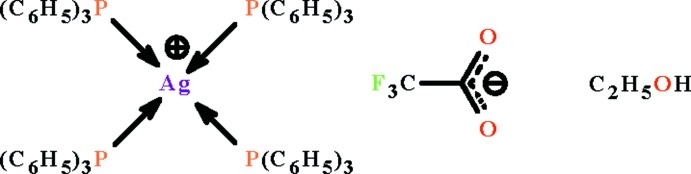


## Experimental

### 

#### Crystal data

[Ag(C_18_H_15_P)_4_](C_2_F_3_O)·C_2_H_6_O
*M* *_r_* = 1316.04
Triclinic, 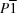

*a* = 11.8005 (2) Å
*b* = 14.5169 (2) Å
*c* = 18.4830 (3) Å
α = 89.4032 (8)°
β = 85.4648 (9)°
γ = 87.7804 (9)°
*V* = 3153.89 (9) Å^3^
*Z* = 2
Mo *K*α radiation
μ = 0.48 mm^−1^
*T* = 100 K
0.20 × 0.18 × 0.16 mm

#### Data collection

Bruker SMART APEX diffractometer
Absorption correction: multi-scan (*SADABS*; Bruker, 2009 ▶) *T*_min_ = 0.910, *T*_max_ = 0.927
27139 measured reflections
14387 independent reflections
11357 reflections with *I* > 2σ(*I*)
*R*_int_ = 0.030

#### Refinement

*R*[*F* ^2^ > 2σ(*F* ^2^)] = 0.047
*wR*(*F* ^2^) = 0.122
*S* = 1.02
14387 reflections
868 parameters
218 restraints
H-atom parameters constrained
Δρ_max_ = 1.72 e Å^−3^
Δρ_min_ = −0.87 e Å^−3^

### 

Data collection: *APEX2* (Bruker, 2009 ▶); cell refinement: *SAINT* (Bruker, 2009 ▶); data reduction: *SAINT*; program(s) used to solve structure: *SHELXS97* (Sheldrick, 2008 ▶); program(s) used to refine structure: *SHELXL97* (Sheldrick, 2008 ▶); molecular graphics: *X-SEED* (Barbour, 2001 ▶); software used to prepare material for publication: *publCIF* (Westrip, 2010 ▶).

## Supplementary Material

Crystal structure: contains datablock(s) global, I. DOI: 10.1107/S1600536812045060/xu5640sup1.cif

Structure factors: contains datablock(s) I. DOI: 10.1107/S1600536812045060/xu5640Isup2.hkl

Additional supplementary materials:  crystallographic information; 3D view; checkCIF report

## Figures and Tables

**Table 1:** Hydrogen-bond geometry (Å, °)

*D*—H⋯*A*	*D*—H	H⋯*A*	*D*⋯*A*	*D*—H⋯*A*
O3—H3⋯O1	0.84	2.41	2.728 (8)	104
O3′—H3′⋯O1′	0.84	2.03	2.72 (2)	138

**Fig. 1.**
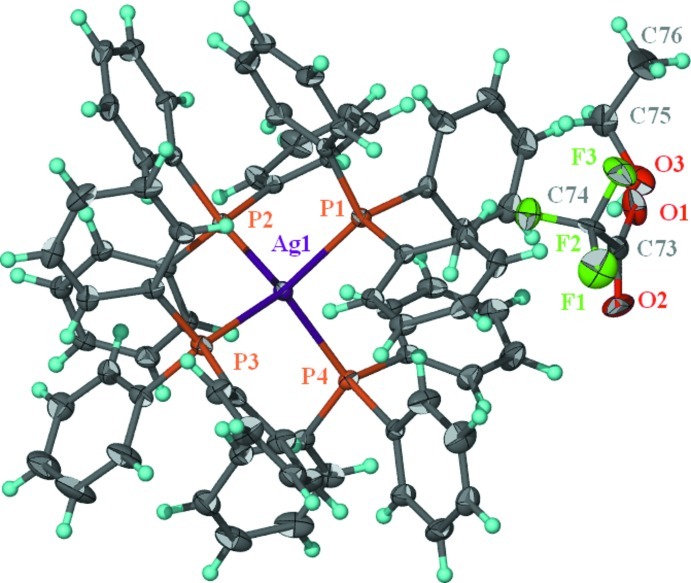
Thermal ellipsoid plot (Barbour, 2001) of [Ag(C18H15P)4](CF3CO2).C2H5OH at the 70% probability level; hydrogen atoms are drawn as spheres of arbitrary radius. The disorder is not shown, and only selected atoms are labeled owing to crowding.

**Fig. 2.**
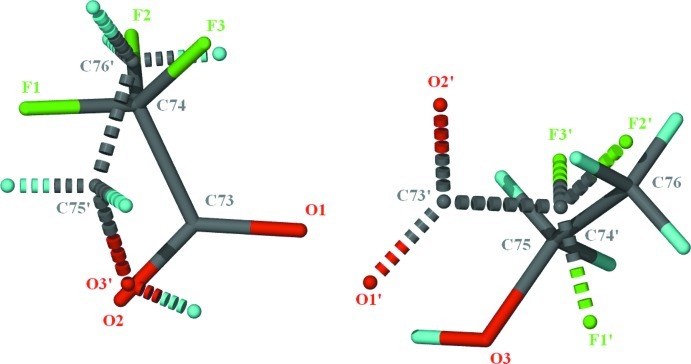
Disorder in the anion/solvent.

## supplementary crystallographic information

### Comment

Silver trifluoroacetate acetate forms a mononuclear bis-adduct with triphenylphosphane when the reaction was carried out in ethanol solvent (Ng, 1998). A similar synthesis but with a 1:4 stoichiometry has yielded a salt (Scheme I, Fig. 1) in which four phosphane ligands bind to the metal atom; the trifluoacetate anion does not engaged in coordination, and is instead disordered with respect to an ethanol molecule. The disorder is such that the anion occupies two positions, as does the two solvent molecules (Fig. 2). The anion and solvent are engaged in weak hydrogen bonding (Table 1).

### Experimental

Silver trifluoroacetate (1 mmol) and triphenylphosphane (4 mmol) were heated in ethanol (25 ml) until the reactants dissolved completely. Colorless crystals were isolated from the filtered solution after several days.

### Refinement

Carbon-bound H-atoms were placed in calculated positions (C—H 0.95 to 0.98 Å) and were included in the refinement in the riding model approximation, with *U*(H) set to 1.2 to 1.5*U*(C).

The silver atom is disordered over two positions in a 0.960 (2): 0.040 ratio. The minor component atom could not be refined anisotropically, and its temperature factor was instead tied to the equivalent isotropic temperature factor of the major component by a factor of one. Neglecting the minor component resulted in largest peaks in vicinity of Ag1.

The trifluoroacetate ion is disordered over two positions; as the occupancy refined to nearly 50%, the disorder was then fixed as a 1:1 type of disorder. The C–O distances were restrained to 1.25±0.01 Å, the C–C distances to 1.50±0.01 Å and the C–F distances to 1.35±0.01 Å. Additionally, the O···O distances were restrained to 2.17±0.01 Å and the F···F distances to 2.21±0.01 Å.

Both half-occupancy trifluoroacetate portions are each hydrogen bonded to a half-occupancy ethanol molecule. For the ethanol molecules, the C–O distance were restrained to 1.45±0.01 Å and the C–C distances to 1.50±0.01 Å. The H atoms were placed in riding positions (O–H 0.84 Å) and their temperature factors tied by a factor of 1.5 times.

The anisotropic temperature factors of the disordered atoms were restrained to be nearly isotropic.

Of the four triphenylphospine groups, two have each a disordered phenyl ring. The disorder was also treated as a 1:1 type of disorder. Pairs of As—C distances were restrained to within 0.01 Å of each other. The aromatic rings were refined as a rigid hexagon of 1.39 Å sides, and the temperature factors of the primed atoms were set to those of the unprimed ones. The anisotropic temperature factors were also restrained to be nearly isotropic.

The (0 - 1 1) reflection that was affected by the beamstop as well as (-5 4 6) were omitted.

The final difference Fourier map was featureless except for a peak at 0.86 Å from Ag1 and a hole at 0.68 Å from the same atom.

### Crystal data

**Table:**

[Ag(C_18_H_15_P)_4_](C_2_F_3_O)·C_2_H_6_O	*Z* = 2
*M**_r_* = 1316.04	*F*(000) = 1360
Triclinic, *P*1	*D*_x_ = 1.386 Mg m^−^^3^
Hall symbol: -P 1	Mo *K*α radiation, λ = 0.71073 Å
*a* = 11.8005 (2) Å	Cell parameters from 7238 reflections
*b* = 14.5169 (2) Å	θ = 2.2–28.1°
*c* = 18.4830 (3) Å	µ = 0.48 mm^−^^1^
α = 89.4032 (8)°	*T* = 100 K
β = 85.4648 (9)°	Prism, colorless
γ = 87.7804 (9)°	0.20 × 0.18 × 0.16 mm
*V* = 3153.89 (9) Å^3^	

### Data collection

**Table:**

Bruker SMART APEX diffractometer	14387 independent reflections
Radiation source: fine-focus sealed tube	11357 reflections with *I* > 2σ(*I*)
Graphite monochromator	*R*_int_ = 0.030
ω scans	θ_max_ = 27.5°, θ_min_ = 1.1°
Absorption correction: multi-scan (*SADABS*; Bruker, 2009)	*h* = −15→10
*T*_min_ = 0.910, *T*_max_ = 0.927	*k* = −18→18
27139 measured reflections	*l* = −24→24

### Refinement

**Table:**

Refinement on *F*^2^	Primary atom site location: structure-invariant direct methods
Least-squares matrix: full	Secondary atom site location: difference Fourier map
*R*[*F*^2^ > 2σ(*F*^2^)] = 0.047	Hydrogen site location: inferred from neighbouring sites
*wR*(*F*^2^) = 0.122	H-atom parameters constrained
*S* = 1.02	*w* = 1/[σ^2^(*F*_o_^2^) + (0.052*P*)^2^ + 3.9561*P*] where *P* = (*F*_o_^2^ + 2*F*_c_^2^)/3
14387 reflections	(Δ/σ)_max_ = 0.002
868 parameters	Δρ_max_ = 1.72 e Å^−^^3^
218 restraints	Δρ_min_ = −0.87 e Å^−^^3^

### Special details

**Table:**

Geometry. All e.s.d.'s (except the e.s.d. in the dihedral angle between two l.s. planes) are estimated using the full covariance matrix. The cell e.s.d.'s are taken into account individually in the estimation of e.s.d.'s in distances, angles and torsion angles; correlations between e.s.d.'s in cell parameters are only used when they are defined by crystal symmetry. An approximate (isotropic) treatment of cell e.s.d.'s is used for estimating e.s.d.'s involving l.s. planes.

### Fractional atomic coordinates and isotropic or equivalent isotropic displacement parameters (Å^2^)

**Table:**

	*x*	*y*	*z*	*U*_iso_*/*U*_eq_	Occ. (<1)
Ag1	0.49426 (2)	0.25264 (3)	0.249511 (12)	0.01994 (10)	0.9595 (15)
Ag1'	0.4729 (6)	0.2070 (7)	0.2557 (3)	0.023 (2)*	0.0405 (15)
P1	0.57160 (7)	0.41762 (5)	0.21736 (4)	0.02088 (16)	
P2	0.34036 (6)	0.21029 (5)	0.16133 (4)	0.02005 (16)	
P3	0.40570 (7)	0.25208 (5)	0.38354 (4)	0.02290 (17)	
P4	0.66173 (7)	0.12645 (5)	0.23472 (4)	0.02154 (16)	
C1	0.6542 (14)	0.4209 (14)	0.1296 (5)	0.0229 (11)	0.50
C2	0.7377 (14)	0.3521 (12)	0.1148 (7)	0.024 (2)	0.50
H2	0.7530	0.3071	0.1507	0.029*	0.50
C3	0.7989 (12)	0.3491 (11)	0.0473 (8)	0.0366 (13)	0.50
H3A	0.8560	0.3021	0.0372	0.044*	0.50
C4	0.7765 (12)	0.4150 (11)	−0.0053 (5)	0.0434 (19)	0.50
H4	0.8183	0.4130	−0.0514	0.052*	0.50
C5	0.6930 (12)	0.4838 (8)	0.0095 (5)	0.040 (2)	0.50
H5	0.6777	0.5289	−0.0265	0.048*	0.50
C6	0.6318 (11)	0.4868 (11)	0.0769 (7)	0.034 (2)	0.50
H6	0.5747	0.5339	0.0870	0.041*	0.50
C1'	0.6453 (13)	0.4243 (14)	0.1265 (5)	0.0229 (11)	0.50
C2'	0.7161 (14)	0.3487 (11)	0.1062 (7)	0.024 (2)	0.50
H2'	0.7162	0.2949	0.1359	0.029*	0.50
C3'	0.7868 (13)	0.3518 (10)	0.0424 (9)	0.0366 (13)	0.50
H3'A	0.8351	0.3002	0.0285	0.044*	0.50
C4'	0.7866 (12)	0.4306 (11)	−0.0010 (6)	0.0434 (19)	0.50
H4'	0.8349	0.4328	−0.0446	0.052*	0.50
C5'	0.7158 (12)	0.5062 (8)	0.0194 (5)	0.040 (2)	0.50
H5'	0.7157	0.5600	−0.0103	0.048*	0.50
C6'	0.6452 (12)	0.5031 (11)	0.0831 (7)	0.034 (2)	0.50
H6'	0.5968	0.5548	0.0970	0.041*	0.50
C7	0.6726 (3)	0.4645 (2)	0.27605 (15)	0.0219 (6)	
C8	0.6548 (3)	0.4498 (2)	0.35090 (16)	0.0259 (6)	
H8	0.5944	0.4130	0.3697	0.031*	
C9	0.7255 (3)	0.4890 (2)	0.39783 (17)	0.0295 (7)	
H9	0.7135	0.4786	0.4486	0.035*	
C10	0.8130 (3)	0.5429 (2)	0.37087 (18)	0.0337 (8)	
H10	0.8601	0.5706	0.4032	0.040*	
C11	0.8325 (3)	0.5568 (2)	0.29674 (19)	0.0344 (8)	
H11	0.8937	0.5930	0.2782	0.041*	
C12	0.7622 (3)	0.5178 (2)	0.24969 (17)	0.0290 (7)	
H12	0.7756	0.5276	0.1989	0.035*	
C13	0.4659 (3)	0.5128 (2)	0.21474 (15)	0.0213 (6)	
C14	0.4809 (3)	0.5999 (2)	0.24330 (16)	0.0273 (7)	
H14	0.5506	0.6130	0.2628	0.033*	
C15	0.3939 (3)	0.6675 (2)	0.24326 (17)	0.0319 (7)	
H15	0.4049	0.7264	0.2629	0.038*	
C16	0.2923 (3)	0.6503 (2)	0.21526 (17)	0.0315 (7)	
H16	0.2333	0.6968	0.2156	0.038*	
C17	0.2770 (3)	0.5642 (3)	0.18652 (19)	0.0357 (8)	
H17	0.2070	0.5516	0.1671	0.043*	
C18	0.3635 (3)	0.4961 (2)	0.18587 (18)	0.0311 (7)	
H18	0.3524	0.4376	0.1655	0.037*	
C19	0.2138 (9)	0.2872 (9)	0.1581 (11)	0.0211 (6)	0.50
C20	0.1725 (12)	0.3355 (9)	0.2198 (9)	0.0259 (19)	0.50
H20	0.2080	0.3268	0.2639	0.031*	0.50
C21	0.0793 (12)	0.3967 (8)	0.2170 (8)	0.031 (2)	0.50
H21	0.0511	0.4298	0.2591	0.037*	0.50
C22	0.0274 (9)	0.4095 (9)	0.1525 (10)	0.0325 (14)	0.50
H22	−0.0362	0.4513	0.1506	0.039*	0.50
C23	0.0687 (11)	0.3611 (8)	0.0908 (9)	0.032 (2)	0.50
H23	0.0333	0.3698	0.0467	0.038*	0.50
C24	0.1619 (11)	0.3000 (7)	0.0936 (9)	0.024 (2)	0.50
H24	0.1901	0.2669	0.0515	0.029*	0.50
C19'	0.2151 (9)	0.2889 (9)	0.1579 (11)	0.0211 (6)	0.50
C20'	0.1617 (12)	0.3165 (9)	0.2243 (9)	0.0259 (19)	0.50
H20B	0.1897	0.2941	0.2682	0.031*	0.50
C21'	0.0672 (12)	0.3770 (8)	0.2264 (8)	0.031 (2)	0.50
H21B	0.0306	0.3959	0.2717	0.037*	0.50
C22'	0.0262 (9)	0.4098 (9)	0.1621 (10)	0.0325 (14)	0.50
H22B	−0.0383	0.4511	0.1636	0.039*	0.50
C23'	0.0797 (11)	0.3821 (8)	0.0958 (8)	0.032 (2)	0.50
H23B	0.0517	0.4045	0.0518	0.038*	0.50
C24'	0.1742 (11)	0.3217 (8)	0.0937 (9)	0.024 (2)	0.50
H24B	0.2107	0.3028	0.0483	0.029*	0.50
C25	0.2782 (3)	0.0978 (2)	0.17701 (14)	0.0210 (6)	
C26	0.3512 (3)	0.0204 (2)	0.18005 (17)	0.0284 (7)	
H26	0.4311	0.0271	0.1731	0.034*	
C27	0.3086 (3)	−0.0657 (2)	0.19307 (18)	0.0331 (7)	
H27	0.3594	−0.1179	0.1947	0.040*	
C28	0.1925 (3)	−0.0769 (2)	0.20376 (16)	0.0304 (7)	
H28	0.1636	−0.1365	0.2125	0.036*	
C29	0.1197 (3)	−0.0012 (2)	0.20163 (18)	0.0328 (7)	
H29	0.0400	−0.0083	0.2095	0.039*	
C30	0.1619 (3)	0.0858 (2)	0.18796 (17)	0.0290 (7)	
H30	0.1106	0.1377	0.1861	0.035*	
C31	0.3928 (3)	0.2059 (2)	0.06605 (15)	0.0213 (6)	
C32	0.3584 (3)	0.1416 (2)	0.01793 (16)	0.0312 (7)	
H32	0.3097	0.0943	0.0353	0.037*	
C33	0.3945 (4)	0.1458 (3)	−0.05523 (18)	0.0418 (9)	
H33	0.3705	0.1016	−0.0878	0.050*	
C34	0.4658 (3)	0.2147 (2)	−0.08077 (18)	0.0380 (8)	
H34	0.4890	0.2186	−0.1311	0.046*	
C35	0.5029 (3)	0.2774 (2)	−0.03357 (18)	0.0328 (7)	
H35	0.5533	0.3235	−0.0511	0.039*	
C36	0.4669 (3)	0.2733 (2)	0.03937 (17)	0.0274 (7)	
H36	0.4928	0.3169	0.0717	0.033*	
C37	0.3416 (3)	0.1458 (2)	0.41606 (16)	0.0272 (7)	
C38	0.2692 (3)	0.1035 (2)	0.37159 (17)	0.0297 (7)	
H38	0.2577	0.1292	0.3251	0.036*	
C39	0.2139 (3)	0.0250 (3)	0.3940 (2)	0.0385 (8)	
H39	0.1649	−0.0032	0.3632	0.046*	
C40	0.2301 (4)	−0.0123 (3)	0.4614 (2)	0.0569 (12)	
H40	0.1910	−0.0657	0.4775	0.068*	
C41	0.3027 (5)	0.0275 (3)	0.5053 (2)	0.0691 (16)	
H41	0.3147	0.0005	0.5513	0.083*	
C42	0.3587 (4)	0.1063 (3)	0.4835 (2)	0.0481 (11)	
H42	0.4086	0.1333	0.5144	0.058*	
C43	0.5011 (3)	0.2772 (2)	0.45307 (15)	0.0250 (6)	
C44	0.6071 (3)	0.2316 (3)	0.45084 (18)	0.0368 (8)	
H44	0.6274	0.1875	0.4140	0.044*	
C45	0.6832 (3)	0.2496 (3)	0.50158 (19)	0.0396 (8)	
H45	0.7549	0.2173	0.4995	0.048*	
C46	0.6564 (3)	0.3134 (2)	0.55461 (18)	0.0356 (8)	
H46	0.7091	0.3256	0.5893	0.043*	
C47	0.5521 (3)	0.3599 (3)	0.55731 (19)	0.0404 (9)	
H47	0.5332	0.4047	0.5939	0.049*	
C48	0.4744 (3)	0.3420 (2)	0.50723 (17)	0.0316 (7)	
H48	0.4026	0.3741	0.5099	0.038*	
C49	0.2877 (3)	0.3360 (2)	0.40079 (15)	0.0235 (6)	
C50	0.1899 (3)	0.3173 (2)	0.44508 (16)	0.0286 (7)	
H50	0.1838	0.2592	0.4688	0.034*	
C51	0.1018 (3)	0.3828 (3)	0.45465 (19)	0.0352 (8)	
H51	0.0355	0.3695	0.4849	0.042*	
C52	0.1103 (3)	0.4672 (3)	0.42015 (18)	0.0370 (8)	
H52	0.0493	0.5119	0.4263	0.044*	
C53	0.2076 (3)	0.4872 (2)	0.37652 (17)	0.0358 (8)	
H53	0.2136	0.5458	0.3533	0.043*	
C54	0.2958 (3)	0.4220 (2)	0.36680 (16)	0.0306 (7)	
H54	0.3623	0.4358	0.3369	0.037*	
C55	0.7365 (3)	0.1113 (2)	0.14455 (16)	0.0242 (6)	
C56	0.6711 (3)	0.1029 (2)	0.08542 (16)	0.0272 (7)	
H56	0.5905	0.1052	0.0928	0.033*	
C57	0.7228 (3)	0.0913 (2)	0.01613 (17)	0.0326 (8)	
H57	0.6776	0.0859	−0.0238	0.039*	
C58	0.8397 (3)	0.0874 (3)	0.00484 (18)	0.0402 (9)	
H58	0.8749	0.0789	−0.0427	0.048*	
C59	0.9056 (3)	0.0961 (3)	0.06287 (19)	0.0457 (10)	
H59	0.9862	0.0933	0.0551	0.055*	
C60	0.8539 (3)	0.1088 (3)	0.13279 (18)	0.0374 (8)	
H60	0.8993	0.1159	0.1724	0.045*	
C61	0.7769 (3)	0.1492 (2)	0.29168 (15)	0.0223 (6)	
C62	0.8057 (3)	0.2397 (3)	0.3006 (2)	0.0424 (9)	
H62	0.7656	0.2878	0.2770	0.051*	
C63	0.8923 (4)	0.2609 (3)	0.3435 (2)	0.0480 (10)	
H63	0.9111	0.3234	0.3490	0.058*	
C64	0.9506 (3)	0.1930 (3)	0.37779 (19)	0.0365 (8)	
H64	1.0098	0.2078	0.4071	0.044*	
C65	0.9229 (3)	0.1031 (3)	0.3696 (2)	0.0421 (9)	
H65	0.9635	0.0554	0.3934	0.051*	
C66	0.8365 (3)	0.0808 (2)	0.32717 (19)	0.0335 (8)	
H66	0.8181	0.0182	0.3223	0.040*	
C67	0.6285 (3)	0.0083 (2)	0.26122 (16)	0.0249 (6)	
C68	0.6938 (3)	−0.0683 (2)	0.23552 (18)	0.0359 (8)	
H68	0.7536	−0.0610	0.1986	0.043*	
C69	0.6714 (4)	−0.1553 (3)	0.2639 (2)	0.0482 (11)	
H69	0.7168	−0.2073	0.2467	0.058*	
C70	0.5837 (4)	−0.1668 (3)	0.3169 (2)	0.0486 (11)	
H70	0.5697	−0.2264	0.3365	0.058*	
C71	0.5168 (4)	−0.0923 (3)	0.3413 (2)	0.0422 (9)	
H71	0.4554	−0.1004	0.3770	0.051*	
C72	0.5393 (3)	−0.0052 (2)	0.31369 (19)	0.0339 (8)	
H72	0.4931	0.0463	0.3309	0.041*	
F1	1.0119 (5)	0.8242 (4)	0.4427 (2)	0.0662 (15)	0.50
F2	0.8570 (3)	0.8000 (3)	0.3933 (2)	0.0501 (12)	0.50
F3	0.9765 (4)	0.8959 (3)	0.3440 (3)	0.0540 (13)	0.50
O1	1.0194 (5)	0.7446 (4)	0.2670 (2)	0.0433 (14)	0.50
O2	1.0651 (9)	0.6753 (7)	0.3659 (4)	0.047 (2)	0.50
O3	1.0003 (5)	0.6425 (4)	0.1458 (3)	0.0512 (15)	0.50
H3	0.9827	0.6210	0.1874	0.077*	0.50
C73	1.0236 (6)	0.7369 (4)	0.3308 (3)	0.0308 (15)	0.50
C74	0.9680 (6)	0.8131 (5)	0.3784 (3)	0.0298 (17)	0.50
C75	0.9010 (7)	0.6860 (6)	0.1174 (5)	0.042 (2)	0.50
H75A	0.8427	0.6986	0.1580	0.051*	0.50
H75B	0.8690	0.6430	0.0838	0.051*	0.50
C76	0.9266 (12)	0.7750 (8)	0.0777 (9)	0.082 (4)	0.50
H76A	0.8569	0.8011	0.0587	0.124*	0.50
H76B	0.9841	0.7630	0.0374	0.124*	0.50
H76C	0.9554	0.8188	0.1112	0.124*	0.50
F1'	0.9865 (10)	0.6689 (10)	0.0857 (9)	0.248 (7)	0.50
F2'	0.9180 (14)	0.8159 (8)	0.0845 (11)	0.234 (8)	0.50
F3'	0.8138 (6)	0.7040 (5)	0.1259 (4)	0.100 (2)	0.50
O1'	1.0300 (6)	0.7086 (5)	0.2197 (5)	0.081 (2)	0.50
O2'	0.9371 (7)	0.8392 (5)	0.1985 (7)	0.118 (3)	0.50
C73'	0.9687 (8)	0.7605 (6)	0.1858 (5)	0.075 (3)	0.50
C74'	0.9189 (8)	0.7355 (8)	0.1171 (5)	0.079 (4)	0.50
O3'	1.0611 (19)	0.6908 (11)	0.3634 (11)	0.130 (7)	0.50
H3'	1.0879	0.6930	0.3199	0.196*	0.50
C75'	1.0495 (14)	0.7819 (10)	0.3921 (8)	0.111 (5)	0.50
H75C	1.1145	0.8160	0.3697	0.133*	0.50
H75D	1.0609	0.7762	0.4445	0.133*	0.50
C76'	0.9469 (17)	0.8426 (14)	0.3863 (12)	0.128 (8)	0.50
H76D	0.9535	0.8986	0.4146	0.192*	0.50
H76E	0.8794	0.8101	0.4052	0.192*	0.50
H76F	0.9398	0.8593	0.3353	0.192*	0.50

### Atomic displacement parameters (Å^2^)

**Table:**

	*U*^11^	*U*^22^	*U*^33^	*U*^12^	*U*^13^	*U*^23^
Ag1	0.02173 (13)	0.0206 (2)	0.01755 (11)	−0.00173 (11)	−0.00135 (8)	−0.00055 (9)
P1	0.0226 (4)	0.0212 (4)	0.0190 (3)	−0.0013 (3)	−0.0026 (3)	−0.0003 (3)
P2	0.0193 (4)	0.0223 (4)	0.0189 (3)	−0.0018 (3)	−0.0026 (3)	−0.0014 (3)
P3	0.0289 (4)	0.0235 (4)	0.0157 (3)	0.0009 (3)	0.0009 (3)	−0.0003 (3)
P4	0.0205 (4)	0.0231 (4)	0.0212 (3)	0.0013 (3)	−0.0038 (3)	−0.0021 (3)
C1	0.019 (2)	0.030 (2)	0.0199 (14)	−0.0040 (18)	−0.0026 (14)	−0.0031 (13)
C2	0.017 (5)	0.029 (2)	0.027 (3)	−0.006 (3)	−0.008 (3)	−0.005 (2)
C3	0.029 (3)	0.046 (2)	0.035 (2)	0.000 (2)	0.000 (2)	−0.0126 (18)
C4	0.044 (3)	0.060 (5)	0.0253 (19)	−0.003 (3)	0.007 (2)	−0.006 (2)
C5	0.053 (5)	0.043 (6)	0.025 (3)	−0.008 (4)	−0.001 (3)	0.007 (3)
C6	0.047 (3)	0.029 (5)	0.024 (2)	0.000 (3)	0.001 (2)	0.001 (2)
C1'	0.019 (2)	0.030 (2)	0.0199 (14)	−0.0040 (18)	−0.0026 (14)	−0.0031 (13)
C2'	0.017 (5)	0.029 (2)	0.027 (3)	−0.006 (3)	−0.008 (3)	−0.005 (2)
C3'	0.029 (3)	0.046 (2)	0.035 (2)	0.000 (2)	0.000 (2)	−0.0126 (18)
C4'	0.044 (3)	0.060 (5)	0.0253 (19)	−0.003 (3)	0.007 (2)	−0.006 (2)
C5'	0.053 (5)	0.043 (6)	0.025 (3)	−0.008 (4)	−0.001 (3)	0.007 (3)
C6'	0.047 (3)	0.029 (5)	0.024 (2)	0.000 (3)	0.001 (2)	0.001 (2)
C7	0.0222 (15)	0.0224 (15)	0.0209 (13)	0.0028 (12)	−0.0023 (11)	−0.0014 (11)
C8	0.0289 (17)	0.0233 (16)	0.0252 (15)	0.0008 (13)	−0.0016 (12)	0.0018 (12)
C9	0.0371 (19)	0.0284 (17)	0.0231 (14)	0.0043 (14)	−0.0057 (13)	−0.0026 (12)
C10	0.0324 (18)	0.0360 (19)	0.0344 (17)	0.0047 (15)	−0.0144 (14)	−0.0105 (14)
C11	0.0240 (17)	0.039 (2)	0.0410 (19)	−0.0080 (15)	−0.0040 (14)	−0.0053 (15)
C12	0.0275 (17)	0.0347 (18)	0.0248 (15)	−0.0040 (14)	−0.0010 (13)	−0.0006 (13)
C13	0.0207 (15)	0.0227 (15)	0.0201 (13)	0.0014 (12)	0.0005 (11)	0.0021 (11)
C14	0.0281 (17)	0.0262 (17)	0.0282 (15)	−0.0018 (13)	−0.0065 (13)	0.0007 (12)
C15	0.040 (2)	0.0235 (17)	0.0315 (16)	0.0071 (14)	−0.0043 (14)	−0.0013 (13)
C16	0.0318 (18)	0.0333 (19)	0.0280 (16)	0.0082 (15)	0.0004 (13)	0.0065 (13)
C17	0.0275 (18)	0.040 (2)	0.0409 (19)	−0.0001 (15)	−0.0117 (15)	0.0047 (15)
C18	0.0331 (18)	0.0279 (17)	0.0338 (17)	−0.0012 (14)	−0.0116 (14)	−0.0021 (13)
C19	0.0187 (14)	0.0195 (15)	0.0252 (14)	−0.0022 (12)	−0.0017 (11)	−0.0002 (11)
C20	0.029 (3)	0.022 (5)	0.027 (2)	−0.005 (3)	−0.002 (2)	−0.001 (3)
C21	0.024 (3)	0.031 (5)	0.036 (4)	0.000 (3)	0.007 (2)	−0.005 (3)
C22	0.0221 (17)	0.0325 (19)	0.042 (4)	0.0040 (14)	0.0013 (18)	−0.0026 (19)
C23	0.028 (3)	0.032 (5)	0.036 (2)	0.003 (3)	−0.0085 (19)	0.001 (3)
C24	0.026 (3)	0.019 (5)	0.0274 (16)	−0.002 (3)	−0.0039 (19)	−0.001 (3)
C19'	0.0187 (14)	0.0195 (15)	0.0252 (14)	−0.0022 (12)	−0.0017 (11)	−0.0002 (11)
C20'	0.029 (3)	0.022 (5)	0.027 (2)	−0.005 (3)	−0.002 (2)	−0.001 (3)
C21'	0.024 (3)	0.031 (5)	0.036 (4)	0.000 (3)	0.007 (2)	−0.005 (3)
C22'	0.0221 (17)	0.0325 (19)	0.042 (4)	0.0040 (14)	0.0013 (18)	−0.0026 (19)
C23'	0.028 (3)	0.032 (5)	0.036 (2)	0.003 (3)	−0.0085 (19)	0.001 (3)
C24'	0.026 (3)	0.019 (5)	0.0274 (16)	−0.002 (3)	−0.0039 (19)	−0.001 (3)
C25	0.0243 (15)	0.0212 (15)	0.0179 (13)	−0.0018 (12)	−0.0036 (11)	−0.0013 (11)
C26	0.0241 (16)	0.0304 (18)	0.0300 (16)	0.0007 (13)	0.0001 (13)	0.0040 (13)
C27	0.0361 (19)	0.0264 (17)	0.0351 (17)	0.0072 (15)	0.0031 (14)	0.0045 (13)
C28	0.041 (2)	0.0241 (17)	0.0264 (15)	−0.0040 (14)	−0.0041 (14)	0.0034 (12)
C29	0.0263 (17)	0.0307 (18)	0.0424 (19)	−0.0059 (14)	−0.0078 (14)	0.0071 (14)
C30	0.0246 (16)	0.0257 (17)	0.0366 (17)	0.0002 (13)	−0.0042 (13)	0.0052 (13)
C31	0.0215 (15)	0.0223 (15)	0.0198 (13)	0.0022 (12)	−0.0011 (11)	0.0012 (11)
C32	0.042 (2)	0.0274 (17)	0.0241 (15)	−0.0072 (15)	−0.0009 (14)	−0.0007 (12)
C33	0.067 (3)	0.035 (2)	0.0221 (16)	−0.0021 (19)	0.0020 (16)	−0.0060 (14)
C34	0.050 (2)	0.036 (2)	0.0252 (16)	0.0091 (17)	0.0111 (15)	0.0043 (14)
C35	0.0285 (17)	0.0321 (18)	0.0358 (17)	0.0051 (14)	0.0058 (14)	0.0106 (14)
C36	0.0224 (16)	0.0271 (17)	0.0323 (16)	−0.0002 (13)	−0.0013 (13)	0.0023 (13)
C37	0.0351 (18)	0.0231 (16)	0.0230 (14)	−0.0004 (13)	0.0001 (13)	0.0001 (12)
C38	0.0309 (18)	0.0339 (18)	0.0236 (15)	−0.0013 (14)	0.0021 (13)	−0.0032 (13)
C39	0.039 (2)	0.035 (2)	0.042 (2)	−0.0059 (16)	−0.0059 (16)	−0.0054 (15)
C40	0.071 (3)	0.039 (2)	0.065 (3)	−0.025 (2)	−0.018 (2)	0.019 (2)
C41	0.104 (4)	0.057 (3)	0.053 (3)	−0.038 (3)	−0.036 (3)	0.033 (2)
C42	0.073 (3)	0.040 (2)	0.0344 (19)	−0.020 (2)	−0.0204 (19)	0.0131 (16)
C43	0.0310 (17)	0.0260 (16)	0.0173 (13)	−0.0009 (13)	0.0025 (12)	0.0029 (11)
C44	0.039 (2)	0.040 (2)	0.0304 (17)	0.0116 (16)	−0.0044 (15)	−0.0064 (14)
C45	0.037 (2)	0.044 (2)	0.0375 (19)	0.0110 (17)	−0.0063 (16)	0.0006 (16)
C46	0.040 (2)	0.038 (2)	0.0304 (17)	−0.0053 (16)	−0.0123 (15)	0.0032 (14)
C47	0.047 (2)	0.045 (2)	0.0313 (17)	0.0012 (18)	−0.0106 (16)	−0.0132 (15)
C48	0.0322 (18)	0.0349 (19)	0.0272 (16)	0.0048 (15)	−0.0022 (13)	−0.0075 (13)
C49	0.0292 (16)	0.0260 (16)	0.0156 (13)	0.0014 (13)	−0.0036 (11)	−0.0040 (11)
C50	0.0290 (17)	0.0337 (18)	0.0232 (14)	−0.0046 (14)	−0.0011 (12)	−0.0061 (12)
C51	0.0276 (18)	0.040 (2)	0.0381 (18)	−0.0029 (15)	−0.0006 (14)	−0.0159 (15)
C52	0.0325 (19)	0.043 (2)	0.0361 (18)	0.0125 (16)	−0.0128 (15)	−0.0194 (16)
C53	0.051 (2)	0.0303 (19)	0.0259 (16)	0.0084 (16)	−0.0092 (15)	−0.0037 (13)
C54	0.040 (2)	0.0304 (18)	0.0204 (14)	0.0022 (15)	0.0003 (13)	0.0024 (12)
C55	0.0268 (16)	0.0225 (16)	0.0233 (14)	0.0049 (13)	−0.0043 (12)	−0.0006 (11)
C56	0.0291 (17)	0.0240 (16)	0.0290 (15)	0.0037 (13)	−0.0087 (13)	−0.0021 (12)
C57	0.046 (2)	0.0299 (18)	0.0221 (15)	0.0065 (15)	−0.0087 (14)	−0.0029 (12)
C58	0.049 (2)	0.046 (2)	0.0235 (16)	0.0147 (18)	0.0008 (15)	−0.0045 (14)
C59	0.031 (2)	0.072 (3)	0.0317 (18)	0.0142 (19)	0.0047 (15)	−0.0032 (18)
C60	0.0298 (18)	0.058 (2)	0.0246 (16)	0.0061 (17)	−0.0047 (14)	−0.0022 (15)
C61	0.0196 (15)	0.0275 (16)	0.0197 (13)	−0.0004 (12)	−0.0009 (11)	−0.0028 (11)
C62	0.048 (2)	0.0277 (19)	0.054 (2)	−0.0013 (17)	−0.0228 (19)	−0.0033 (16)
C63	0.049 (2)	0.035 (2)	0.063 (3)	−0.0093 (18)	−0.018 (2)	−0.0127 (18)
C64	0.0283 (18)	0.046 (2)	0.0370 (18)	−0.0094 (16)	−0.0096 (14)	−0.0072 (15)
C65	0.034 (2)	0.048 (2)	0.048 (2)	−0.0059 (17)	−0.0220 (17)	0.0087 (17)
C66	0.0306 (18)	0.0302 (18)	0.0422 (19)	−0.0081 (15)	−0.0153 (15)	0.0062 (14)
C67	0.0274 (16)	0.0254 (16)	0.0232 (14)	−0.0024 (13)	−0.0094 (12)	−0.0021 (12)
C68	0.052 (2)	0.0288 (18)	0.0280 (16)	0.0021 (16)	−0.0080 (15)	−0.0054 (13)
C69	0.079 (3)	0.0255 (19)	0.042 (2)	0.0049 (19)	−0.020 (2)	−0.0077 (15)
C70	0.078 (3)	0.033 (2)	0.039 (2)	−0.020 (2)	−0.025 (2)	0.0074 (16)
C71	0.045 (2)	0.044 (2)	0.040 (2)	−0.0138 (18)	−0.0113 (17)	0.0101 (16)
C72	0.0297 (18)	0.0336 (19)	0.0388 (18)	−0.0013 (15)	−0.0059 (14)	0.0044 (14)
F1	0.076 (4)	0.085 (4)	0.040 (3)	−0.006 (3)	−0.018 (2)	−0.027 (2)
F2	0.035 (2)	0.062 (3)	0.050 (3)	0.007 (2)	0.013 (2)	0.001 (2)
F3	0.074 (3)	0.020 (2)	0.064 (3)	0.005 (2)	0.015 (3)	0.008 (2)
O1	0.047 (3)	0.063 (4)	0.020 (2)	−0.023 (3)	0.006 (2)	−0.012 (2)
O2	0.042 (4)	0.042 (4)	0.054 (4)	0.024 (3)	−0.003 (3)	0.006 (3)
O3	0.065 (4)	0.040 (3)	0.044 (3)	0.012 (3)	0.013 (3)	0.001 (2)
C73	0.026 (3)	0.022 (3)	0.045 (4)	−0.007 (3)	−0.003 (3)	0.005 (3)
C74	0.046 (4)	0.023 (4)	0.022 (3)	−0.003 (3)	−0.009 (3)	0.010 (3)
C75	0.042 (5)	0.056 (5)	0.029 (4)	−0.010 (4)	−0.001 (3)	−0.009 (4)
C76	0.068 (6)	0.043 (5)	0.141 (9)	−0.035 (5)	−0.023 (6)	0.052 (6)
F1'	0.232 (10)	0.293 (11)	0.217 (10)	0.045 (9)	−0.015 (8)	−0.056 (9)
F2'	0.217 (11)	0.239 (11)	0.249 (11)	−0.049 (9)	−0.028 (8)	0.065 (9)
F3'	0.080 (5)	0.128 (6)	0.095 (5)	−0.025 (4)	−0.008 (4)	−0.014 (4)
O1'	0.053 (4)	0.085 (5)	0.105 (6)	0.009 (4)	−0.011 (4)	0.035 (4)
O2'	0.061 (5)	0.058 (5)	0.233 (9)	0.004 (4)	−0.004 (5)	0.011 (5)
C73'	0.046 (5)	0.079 (6)	0.099 (7)	−0.012 (5)	−0.002 (5)	0.014 (6)
C74'	0.059 (6)	0.120 (9)	0.058 (6)	0.010 (7)	−0.011 (5)	−0.018 (7)
O3'	0.120 (10)	0.105 (10)	0.165 (11)	0.001 (8)	−0.007 (8)	−0.010 (8)
C75'	0.131 (9)	0.111 (9)	0.091 (8)	−0.006 (8)	−0.010 (7)	−0.007 (7)
C76'	0.135 (11)	0.119 (11)	0.125 (10)	0.046 (8)	0.007 (8)	−0.014 (8)

### Geometric parameters (Å, º)

**Table:**

Ag1—P3	2.6115 (8)	C33—C34	1.387 (5)
Ag1—P2	2.6283 (8)	C33—H33	0.9500
Ag1—P1	2.6413 (9)	C34—C35	1.374 (5)
Ag1—P4	2.6421 (8)	C34—H34	0.9500
Ag1'—P2	2.433 (6)	C35—C36	1.382 (4)
Ag1'—P4	2.480 (6)	C35—H35	0.9500
Ag1'—P3	2.517 (6)	C36—H36	0.9500
P1—C1	1.827 (5)	C37—C42	1.392 (4)
P1—C7	1.828 (3)	C37—C38	1.395 (4)
P1—C13	1.829 (3)	C38—C39	1.378 (5)
P1—C1'	1.832 (5)	C38—H38	0.9500
P2—C31	1.821 (3)	C39—C40	1.376 (5)
P2—C25	1.826 (3)	C39—H39	0.9500
P2—C19	1.834 (5)	C40—C41	1.373 (6)
P2—C19'	1.836 (5)	C40—H40	0.9500
P3—C37	1.821 (3)	C41—C42	1.384 (6)
P3—C43	1.823 (3)	C41—H41	0.9500
P3—C49	1.825 (3)	C42—H42	0.9500
P4—C61	1.826 (3)	C43—C44	1.390 (5)
P4—C67	1.828 (3)	C43—C48	1.390 (4)
P4—C55	1.834 (3)	C44—C45	1.383 (5)
C1—C2	1.3900	C44—H44	0.9500
C1—C6	1.3900	C45—C46	1.366 (5)
C2—C3	1.3900	C45—H45	0.9500
C2—H2	0.9500	C46—C47	1.379 (5)
C3—C4	1.3900	C46—H46	0.9500
C3—H3A	0.9500	C47—C48	1.386 (5)
C4—C5	1.3900	C47—H47	0.9500
C4—H4	0.9500	C48—H48	0.9500
C5—C6	1.3900	C49—C54	1.395 (4)
C5—H5	0.9500	C49—C50	1.395 (4)
C6—H6	0.9500	C50—C51	1.383 (5)
C1'—C2'	1.3900	C50—H50	0.9500
C1'—C6'	1.3900	C51—C52	1.380 (5)
C2'—C3'	1.3900	C51—H51	0.9500
C2'—H2'	0.9500	C52—C53	1.389 (5)
C3'—C4'	1.3900	C52—H52	0.9500
C3'—H3'A	0.9500	C53—C54	1.382 (5)
C4'—C5'	1.3900	C53—H53	0.9500
C4'—H4'	0.9500	C54—H54	0.9500
C5'—C6'	1.3900	C55—C60	1.384 (5)
C5'—H5'	0.9500	C55—C56	1.395 (4)
C6'—H6'	0.9500	C56—C57	1.383 (4)
C7—C12	1.389 (4)	C56—H56	0.9500
C7—C8	1.398 (4)	C57—C58	1.377 (5)
C8—C9	1.391 (4)	C57—H57	0.9500
C8—H8	0.9500	C58—C59	1.384 (5)
C9—C10	1.378 (5)	C58—H58	0.9500
C9—H9	0.9500	C59—C60	1.396 (5)
C10—C11	1.385 (5)	C59—H59	0.9500
C10—H10	0.9500	C60—H60	0.9500
C11—C12	1.387 (4)	C61—C62	1.384 (5)
C11—H11	0.9500	C61—C66	1.384 (4)
C12—H12	0.9500	C62—C63	1.389 (5)
C13—C18	1.390 (4)	C62—H62	0.9500
C13—C14	1.397 (4)	C63—C64	1.360 (5)
C14—C15	1.393 (5)	C63—H63	0.9500
C14—H14	0.9500	C64—C65	1.371 (5)
C15—C16	1.376 (5)	C64—H64	0.9500
C15—H15	0.9500	C65—C66	1.384 (5)
C16—C17	1.387 (5)	C65—H65	0.9500
C16—H16	0.9500	C66—H66	0.9500
C17—C18	1.393 (5)	C67—C72	1.391 (5)
C17—H17	0.9500	C67—C68	1.394 (5)
C18—H18	0.9500	C68—C69	1.390 (5)
C19—C20	1.3900	C68—H68	0.9500
C19—C24	1.3900	C69—C70	1.381 (6)
C20—C21	1.3900	C69—H69	0.9500
C20—H20	0.9500	C70—C71	1.373 (6)
C21—C22	1.3900	C70—H70	0.9500
C21—H21	0.9500	C71—C72	1.387 (5)
C22—C23	1.3900	C71—H71	0.9500
C22—H22	0.9500	C72—H72	0.9500
C23—C24	1.3900	F1—C74	1.347 (6)
C23—H23	0.9500	F2—C74	1.337 (7)
C24—H24	0.9500	F3—C74	1.357 (7)
C19'—C20'	1.3900	O1—C73	1.189 (6)
C19'—C24'	1.3900	O2—C73	1.211 (8)
C20'—C21'	1.3900	O3—C75	1.442 (8)
C20'—H20B	0.9500	O3—H3	0.8400
C21'—C22'	1.3900	C73—C74	1.516 (8)
C21'—H21B	0.9500	C75—C76	1.509 (8)
C22'—C23'	1.3900	C75—H75A	0.9900
C22'—H22B	0.9500	C75—H75B	0.9900
C23'—C24'	1.3900	C76—H76A	0.9800
C23'—H23B	0.9500	C76—H76B	0.9800
C24'—H24B	0.9500	C76—H76C	0.9800
C25—C30	1.390 (4)	F1'—C74'	1.337 (9)
C25—C26	1.392 (4)	F2'—C74'	1.307 (9)
C26—C27	1.377 (5)	F3'—C74'	1.336 (8)
C26—H26	0.9500	O1'—C73'	1.226 (8)
C27—C28	1.385 (5)	O2'—C73'	1.209 (8)
C27—H27	0.9500	C73'—C74'	1.495 (9)
C28—C29	1.371 (5)	O3'—C75'	1.428 (10)
C28—H28	0.9500	O3'—H3'	0.8400
C29—C30	1.389 (4)	C75'—C76'	1.480 (10)
C29—H29	0.9500	C75'—H75C	0.9900
C30—H30	0.9500	C75'—H75D	0.9900
C31—C32	1.390 (4)	C76'—H76D	0.9800
C31—C36	1.398 (4)	C76'—H76E	0.9800
C32—C33	1.387 (4)	C76'—H76F	0.9800
C32—H32	0.9500		
			
P3—Ag1—P2	109.99 (3)	C29—C30—C25	120.8 (3)
P3—Ag1—P1	109.06 (3)	C29—C30—H30	119.6
P2—Ag1—P1	110.41 (3)	C25—C30—H30	119.6
P3—Ag1—P4	109.40 (3)	C32—C31—C36	118.5 (3)
P2—Ag1—P4	107.76 (3)	C32—C31—P2	123.2 (2)
P1—Ag1—P4	110.21 (3)	C36—C31—P2	118.2 (2)
P2—Ag1'—P4	120.1 (2)	C33—C32—C31	120.7 (3)
P2—Ag1'—P3	120.2 (3)	C33—C32—H32	119.7
P4—Ag1'—P3	118.2 (2)	C31—C32—H32	119.7
C1—P1—C7	100.7 (5)	C32—C33—C34	119.8 (3)
C1—P1—C13	105.4 (6)	C32—C33—H33	120.1
C7—P1—C13	101.46 (14)	C34—C33—H33	120.1
C7—P1—C1'	103.6 (5)	C35—C34—C33	120.2 (3)
C13—P1—C1'	101.9 (7)	C35—C34—H34	119.9
C1—P1—Ag1	112.6 (6)	C33—C34—H34	119.9
C7—P1—Ag1	117.80 (10)	C34—C35—C36	120.0 (3)
C13—P1—Ag1	116.82 (10)	C34—C35—H35	120.0
C1'—P1—Ag1	113.2 (6)	C36—C35—H35	120.0
C31—P2—C25	103.04 (13)	C35—C36—C31	120.7 (3)
C31—P2—C19	101.7 (6)	C35—C36—H36	119.6
C25—P2—C19	102.2 (5)	C31—C36—H36	119.6
C31—P2—C19'	101.5 (6)	C42—C37—C38	118.6 (3)
C25—P2—C19'	103.1 (5)	C42—C37—P3	123.5 (3)
C31—P2—Ag1'	120.35 (19)	C38—C37—P3	117.9 (2)
C25—P2—Ag1'	99.8 (3)	C39—C38—C37	121.0 (3)
C19—P2—Ag1'	125.9 (6)	C39—C38—H38	119.5
C19'—P2—Ag1'	125.5 (6)	C37—C38—H38	119.5
C31—P2—Ag1	114.19 (10)	C40—C39—C38	119.7 (3)
C25—P2—Ag1	115.59 (9)	C40—C39—H39	120.2
C19—P2—Ag1	118.0 (6)	C38—C39—H39	120.2
C19'—P2—Ag1	117.4 (6)	C41—C40—C39	120.1 (4)
C37—P3—C43	103.28 (14)	C41—C40—H40	120.0
C37—P3—C49	101.67 (15)	C39—C40—H40	120.0
C43—P3—C49	103.48 (14)	C40—C41—C42	120.9 (4)
C37—P3—Ag1'	100.4 (3)	C40—C41—H41	119.6
C43—P3—Ag1'	123.70 (19)	C42—C41—H41	119.6
C49—P3—Ag1'	120.55 (18)	C41—C42—C37	119.7 (4)
C37—P3—Ag1	116.44 (10)	C41—C42—H42	120.1
C43—P3—Ag1	116.33 (10)	C37—C42—H42	120.1
C49—P3—Ag1	113.73 (9)	C44—C43—C48	118.2 (3)
C61—P4—C67	101.70 (14)	C44—C43—P3	118.7 (2)
C61—P4—C55	102.81 (14)	C48—C43—P3	123.1 (3)
C67—P4—C55	102.49 (14)	C45—C44—C43	120.8 (3)
C61—P4—Ag1'	121.0 (2)	C45—C44—H44	119.6
C67—P4—Ag1'	101.6 (3)	C43—C44—H44	119.6
C55—P4—Ag1'	123.51 (17)	C46—C45—C44	120.7 (3)
C61—P4—Ag1	112.37 (10)	C46—C45—H45	119.6
C67—P4—Ag1	117.24 (11)	C44—C45—H45	119.6
C55—P4—Ag1	118.02 (10)	C45—C46—C47	119.3 (3)
C2—C1—C6	120.0	C45—C46—H46	120.4
C2—C1—P1	117.9 (11)	C47—C46—H46	120.4
C6—C1—P1	122.0 (11)	C46—C47—C48	120.7 (3)
C1—C2—C3	120.0	C46—C47—H47	119.7
C1—C2—H2	120.0	C48—C47—H47	119.7
C3—C2—H2	120.0	C47—C48—C43	120.4 (3)
C2—C3—C4	120.0	C47—C48—H48	119.8
C2—C3—H3A	120.0	C43—C48—H48	119.8
C4—C3—H3A	120.0	C54—C49—C50	119.1 (3)
C5—C4—C3	120.0	C54—C49—P3	117.9 (2)
C5—C4—H4	120.0	C50—C49—P3	123.0 (2)
C3—C4—H4	120.0	C51—C50—C49	120.5 (3)
C4—C5—C6	120.0	C51—C50—H50	119.8
C4—C5—H5	120.0	C49—C50—H50	119.8
C6—C5—H5	120.0	C52—C51—C50	119.9 (3)
C5—C6—C1	120.0	C52—C51—H51	120.0
C5—C6—H6	120.0	C50—C51—H51	120.0
C1—C6—H6	120.0	C51—C52—C53	120.2 (3)
C2'—C1'—C6'	120.0	C51—C52—H52	119.9
C2'—C1'—P1	115.6 (11)	C53—C52—H52	119.9
C6'—C1'—P1	123.9 (11)	C54—C53—C52	120.0 (3)
C1'—C2'—C3'	120.0	C54—C53—H53	120.0
C1'—C2'—H2'	120.0	C52—C53—H53	120.0
C3'—C2'—H2'	120.0	C53—C54—C49	120.3 (3)
C2'—C3'—C4'	120.0	C53—C54—H54	119.9
C2'—C3'—H3'A	120.0	C49—C54—H54	119.9
C4'—C3'—H3'A	120.0	C60—C55—C56	119.0 (3)
C3'—C4'—C5'	120.0	C60—C55—P4	123.0 (2)
C3'—C4'—H4'	120.0	C56—C55—P4	118.0 (2)
C5'—C4'—H4'	120.0	C57—C56—C55	120.5 (3)
C6'—C5'—C4'	120.0	C57—C56—H56	119.7
C6'—C5'—H5'	120.0	C55—C56—H56	119.7
C4'—C5'—H5'	120.0	C58—C57—C56	120.2 (3)
C5'—C6'—C1'	120.0	C58—C57—H57	119.9
C5'—C6'—H6'	120.0	C56—C57—H57	119.9
C1'—C6'—H6'	120.0	C57—C58—C59	119.9 (3)
C12—C7—C8	118.9 (3)	C57—C58—H58	120.0
C12—C7—P1	122.7 (2)	C59—C58—H58	120.0
C8—C7—P1	118.3 (2)	C58—C59—C60	120.1 (3)
C9—C8—C7	120.1 (3)	C58—C59—H59	120.0
C9—C8—H8	119.9	C60—C59—H59	120.0
C7—C8—H8	119.9	C55—C60—C59	120.2 (3)
C10—C9—C8	120.2 (3)	C55—C60—H60	119.9
C10—C9—H9	119.9	C59—C60—H60	119.9
C8—C9—H9	119.9	C62—C61—C66	118.0 (3)
C9—C10—C11	120.2 (3)	C62—C61—P4	118.4 (2)
C9—C10—H10	119.9	C66—C61—P4	123.5 (2)
C11—C10—H10	119.9	C61—C62—C63	120.8 (3)
C10—C11—C12	119.8 (3)	C61—C62—H62	119.6
C10—C11—H11	120.1	C63—C62—H62	119.6
C12—C11—H11	120.1	C64—C63—C62	120.5 (4)
C11—C12—C7	120.7 (3)	C64—C63—H63	119.7
C11—C12—H12	119.6	C62—C63—H63	119.7
C7—C12—H12	119.6	C63—C64—C65	119.3 (3)
C18—C13—C14	118.6 (3)	C63—C64—H64	120.3
C18—C13—P1	118.0 (2)	C65—C64—H64	120.3
C14—C13—P1	123.3 (2)	C64—C65—C66	120.8 (3)
C15—C14—C13	120.1 (3)	C64—C65—H65	119.6
C15—C14—H14	119.9	C66—C65—H65	119.6
C13—C14—H14	119.9	C65—C66—C61	120.5 (3)
C16—C15—C14	121.0 (3)	C65—C66—H66	119.8
C16—C15—H15	119.5	C61—C66—H66	119.8
C14—C15—H15	119.5	C72—C67—C68	118.5 (3)
C15—C16—C17	119.1 (3)	C72—C67—P4	118.3 (2)
C15—C16—H16	120.4	C68—C67—P4	122.9 (3)
C17—C16—H16	120.4	C69—C68—C67	120.0 (4)
C16—C17—C18	120.4 (3)	C69—C68—H68	120.0
C16—C17—H17	119.8	C67—C68—H68	120.0
C18—C17—H17	119.8	C70—C69—C68	120.5 (4)
C13—C18—C17	120.6 (3)	C70—C69—H69	119.8
C13—C18—H18	119.7	C68—C69—H69	119.8
C17—C18—H18	119.7	C71—C70—C69	120.2 (4)
C20—C19—C24	120.0	C71—C70—H70	119.9
C20—C19—P2	119.7 (10)	C69—C70—H70	119.9
C24—C19—P2	120.3 (10)	C70—C71—C72	119.6 (4)
C19—C20—C21	120.0	C70—C71—H71	120.2
C19—C20—H20	120.0	C72—C71—H71	120.2
C21—C20—H20	120.0	C71—C72—C67	121.2 (3)
C22—C21—C20	120.0	C71—C72—H72	119.4
C22—C21—H21	120.0	C67—C72—H72	119.4
C20—C21—H21	120.0	O1—C73—O2	129.9 (7)
C23—C22—C21	120.0	O1—C73—C74	117.8 (6)
C23—C22—H22	120.0	O2—C73—C74	112.3 (6)
C21—C22—H22	120.0	F2—C74—F1	106.4 (5)
C22—C23—C24	120.0	F2—C74—F3	106.7 (6)
C22—C23—H23	120.0	F1—C74—F3	105.5 (5)
C24—C23—H23	120.0	F2—C74—C73	111.3 (5)
C23—C24—C19	120.0	F1—C74—C73	115.8 (6)
C23—C24—H24	120.0	F3—C74—C73	110.5 (5)
C19—C24—H24	120.0	O3—C75—C76	112.7 (8)
C20'—C19'—C24'	120.0	O3—C75—H75A	109.0
C20'—C19'—P2	116.4 (10)	C76—C75—H75A	109.0
C24'—C19'—P2	123.6 (10)	O3—C75—H75B	109.0
C21'—C20'—C19'	120.0	C76—C75—H75B	109.0
C21'—C20'—H20B	120.0	H75A—C75—H75B	107.8
C19'—C20'—H20B	120.0	C75—C76—H76A	109.5
C20'—C21'—C22'	120.0	C75—C76—H76B	109.5
C20'—C21'—H21B	120.0	H76A—C76—H76B	109.5
C22'—C21'—H21B	120.0	C75—C76—H76C	109.5
C21'—C22'—C23'	120.0	H76A—C76—H76C	109.5
C21'—C22'—H22B	120.0	H76B—C76—H76C	109.5
C23'—C22'—H22B	120.0	O2'—C73'—O1'	129.4 (10)
C24'—C23'—C22'	120.0	O2'—C73'—C74'	106.2 (9)
C24'—C23'—H23B	120.0	O1'—C73'—C74'	124.4 (10)
C22'—C23'—H23B	120.0	F2'—C74'—F3'	110.5 (9)
C23'—C24'—C19'	120.0	F2'—C74'—F1'	116.9 (10)
C23'—C24'—H24B	120.0	F3'—C74'—F1'	107.6 (8)
C19'—C24'—H24B	120.0	F2'—C74'—C73'	99.9 (12)
C30—C25—C26	118.2 (3)	F3'—C74'—C73'	114.8 (9)
C30—C25—P2	123.3 (2)	F1'—C74'—C73'	107.2 (11)
C26—C25—P2	118.4 (2)	C75'—O3'—H3'	109.5
C27—C26—C25	120.6 (3)	O3'—C75'—C76'	123.3 (16)
C27—C26—H26	119.7	O3'—C75'—H75C	106.5
C25—C26—H26	119.7	C76'—C75'—H75C	106.5
C26—C27—C28	120.7 (3)	O3'—C75'—H75D	106.5
C26—C27—H27	119.6	C76'—C75'—H75D	106.5
C28—C27—H27	119.6	H75C—C75'—H75D	106.5
C29—C28—C27	119.3 (3)	C75'—C76'—H76D	109.5
C29—C28—H28	120.3	C75'—C76'—H76E	109.5
C27—C28—H28	120.3	H76D—C76'—H76E	109.5
C28—C29—C30	120.4 (3)	C75'—C76'—H76F	109.5
C28—C29—H29	119.8	H76D—C76'—H76F	109.5
C30—C29—H29	119.8	H76E—C76'—H76F	109.5
			
P3—Ag1—P1—C1	171.4 (6)	C19—P2—C19'—C20'	83 (76)
P2—Ag1—P1—C1	−67.6 (6)	Ag1'—P2—C19'—C20'	−31.3 (9)
P4—Ag1—P1—C1	51.3 (6)	Ag1—P2—C19'—C20'	−47.3 (7)
P3—Ag1—P1—C7	54.87 (11)	C31—P2—C19'—C24'	6.7 (8)
P2—Ag1—P1—C7	175.83 (11)	C25—P2—C19'—C24'	−99.8 (7)
P4—Ag1—P1—C7	−65.24 (11)	C19—P2—C19'—C24'	−98 (76)
P3—Ag1—P1—C13	−66.35 (10)	Ag1'—P2—C19'—C24'	147.8 (6)
P2—Ag1—P1—C13	54.61 (10)	Ag1—P2—C19'—C24'	131.8 (6)
P4—Ag1—P1—C13	173.54 (10)	C24'—C19'—C20'—C21'	0.0
P3—Ag1—P1—C1'	175.8 (6)	P2—C19'—C20'—C21'	179.1 (11)
P2—Ag1—P1—C1'	−63.3 (6)	C19'—C20'—C21'—C22'	0.0
P4—Ag1—P1—C1'	55.7 (6)	C20'—C21'—C22'—C23'	0.0
P4—Ag1'—P2—C31	−29.2 (6)	C21'—C22'—C23'—C24'	0.0
P3—Ag1'—P2—C31	164.8 (3)	C22'—C23'—C24'—C19'	0.0
P4—Ag1'—P2—C25	82.3 (5)	C20'—C19'—C24'—C23'	0.0
P3—Ag1'—P2—C25	−83.7 (5)	P2—C19'—C24'—C23'	−179.1 (11)
P4—Ag1'—P2—C19	−164.8 (7)	C31—P2—C25—C30	−109.2 (3)
P3—Ag1'—P2—C19	29.2 (9)	C19—P2—C25—C30	−4.0 (7)
P4—Ag1'—P2—C19'	−163.7 (7)	C19'—P2—C25—C30	−3.9 (7)
P3—Ag1'—P2—C19'	30.3 (9)	Ag1'—P2—C25—C30	126.3 (3)
P4—Ag1'—P2—Ag1	−100.2 (7)	Ag1—P2—C25—C30	125.5 (2)
P3—Ag1'—P2—Ag1	93.8 (7)	C31—P2—C25—C26	72.5 (2)
P3—Ag1—P2—C31	−178.70 (11)	C19—P2—C25—C26	177.8 (7)
P1—Ag1—P2—C31	60.90 (11)	C19'—P2—C25—C26	177.8 (7)
P4—Ag1—P2—C31	−59.51 (11)	Ag1'—P2—C25—C26	−52.0 (3)
P3—Ag1—P2—C25	−59.40 (11)	Ag1—P2—C25—C26	−52.7 (2)
P1—Ag1—P2—C25	−179.80 (11)	C30—C25—C26—C27	0.5 (4)
P4—Ag1—P2—C25	59.79 (11)	P2—C25—C26—C27	178.8 (2)
P3—Ag1—P2—C19	61.9 (6)	C25—C26—C27—C28	−0.3 (5)
P1—Ag1—P2—C19	−58.5 (6)	C26—C27—C28—C29	−0.3 (5)
P4—Ag1—P2—C19	−178.9 (6)	C27—C28—C29—C30	0.8 (5)
P3—Ag1—P2—C19'	62.8 (6)	C28—C29—C30—C25	−0.7 (5)
P1—Ag1—P2—C19'	−57.7 (6)	C26—C25—C30—C29	0.1 (5)
P4—Ag1—P2—C19'	−178.1 (6)	P2—C25—C30—C29	−178.2 (2)
P3—Ag1—P2—Ag1'	−62.2 (5)	C25—P2—C31—C32	16.6 (3)
P1—Ag1—P2—Ag1'	177.4 (5)	C19—P2—C31—C32	−89.1 (6)
P4—Ag1—P2—Ag1'	57.0 (5)	C19'—P2—C31—C32	−90.0 (6)
P2—Ag1'—P3—C37	80.6 (5)	Ag1'—P2—C31—C32	126.3 (4)
P4—Ag1'—P3—C37	−85.6 (4)	Ag1—P2—C31—C32	142.7 (2)
P2—Ag1'—P3—C43	−165.6 (3)	C25—P2—C31—C36	−166.7 (2)
P4—Ag1'—P3—C43	28.1 (6)	C19—P2—C31—C36	87.6 (6)
P2—Ag1'—P3—C49	−29.6 (6)	C19'—P2—C31—C36	86.7 (6)
P4—Ag1'—P3—C49	164.1 (3)	Ag1'—P2—C31—C36	−57.0 (4)
P2—Ag1'—P3—Ag1	−98.5 (7)	Ag1—P2—C31—C36	−40.6 (3)
P4—Ag1'—P3—Ag1	95.2 (7)	C36—C31—C32—C33	−1.6 (5)
P2—Ag1—P3—C37	56.35 (13)	P2—C31—C32—C33	175.1 (3)
P1—Ag1—P3—C37	177.57 (12)	C31—C32—C33—C34	0.1 (6)
P4—Ag1—P3—C37	−61.82 (13)	C32—C33—C34—C35	1.6 (6)
P2—Ag1—P3—C43	178.52 (11)	C33—C34—C35—C36	−1.7 (5)
P1—Ag1—P3—C43	−60.27 (12)	C34—C35—C36—C31	0.1 (5)
P4—Ag1—P3—C43	60.34 (12)	C32—C31—C36—C35	1.6 (5)
P2—Ag1—P3—C49	−61.37 (11)	P2—C31—C36—C35	−175.3 (2)
P1—Ag1—P3—C49	59.85 (11)	C43—P3—C37—C42	6.6 (4)
P4—Ag1—P3—C49	−179.54 (11)	C49—P3—C37—C42	−100.5 (3)
P2—Ag1—P3—Ag1'	57.3 (5)	Ag1'—P3—C37—C42	135.1 (4)
P1—Ag1—P3—Ag1'	178.5 (5)	Ag1—P3—C37—C42	135.4 (3)
P4—Ag1—P3—Ag1'	−60.9 (5)	C43—P3—C37—C38	−175.3 (3)
P2—Ag1'—P4—C61	162.5 (3)	C49—P3—C37—C38	77.7 (3)
P3—Ag1'—P4—C61	−31.2 (6)	Ag1'—P3—C37—C38	−46.7 (3)
P2—Ag1'—P4—C67	−86.2 (4)	Ag1—P3—C37—C38	−46.5 (3)
P3—Ag1'—P4—C67	80.1 (4)	C42—C37—C38—C39	1.0 (5)
P2—Ag1'—P4—C55	27.4 (6)	P3—C37—C38—C39	−177.2 (3)
P3—Ag1'—P4—C55	−166.3 (3)	C37—C38—C39—C40	0.1 (6)
P2—Ag1'—P4—Ag1	101.8 (7)	C38—C39—C40—C41	−1.3 (7)
P3—Ag1'—P4—Ag1	−91.9 (6)	C39—C40—C41—C42	1.3 (9)
P3—Ag1—P4—C61	−61.85 (11)	C40—C41—C42—C37	−0.2 (8)
P2—Ag1—P4—C61	178.59 (10)	C38—C37—C42—C41	−1.0 (6)
P1—Ag1—P4—C61	58.05 (11)	P3—C37—C42—C41	177.2 (4)
P3—Ag1—P4—C67	55.44 (11)	C37—P3—C43—C44	81.1 (3)
P2—Ag1—P4—C67	−64.12 (11)	C49—P3—C43—C44	−173.2 (3)
P1—Ag1—P4—C67	175.35 (10)	Ag1'—P3—C43—C44	−31.2 (4)
P3—Ag1—P4—C55	178.76 (12)	Ag1—P3—C43—C44	−47.8 (3)
P2—Ag1—P4—C55	59.20 (12)	C37—P3—C43—C48	−100.8 (3)
P1—Ag1—P4—C55	−61.33 (12)	C49—P3—C43—C48	4.9 (3)
P3—Ag1—P4—Ag1'	64.2 (5)	Ag1'—P3—C43—C48	146.9 (4)
P2—Ag1—P4—Ag1'	−55.4 (5)	Ag1—P3—C43—C48	130.3 (3)
P1—Ag1—P4—Ag1'	−175.9 (5)	C48—C43—C44—C45	0.6 (5)
C7—P1—C1—C2	78.4 (8)	P3—C43—C44—C45	178.8 (3)
C13—P1—C1—C2	−176.4 (7)	C43—C44—C45—C46	−0.7 (6)
C1'—P1—C1—C2	−146 (17)	C44—C45—C46—C47	0.1 (6)
Ag1—P1—C1—C2	−48.0 (10)	C45—C46—C47—C48	0.5 (6)
C7—P1—C1—C6	−104.8 (6)	C46—C47—C48—C43	−0.5 (6)
C13—P1—C1—C6	0.3 (8)	C44—C43—C48—C47	0.0 (5)
C1'—P1—C1—C6	30 (16)	P3—C43—C48—C47	−178.1 (3)
Ag1—P1—C1—C6	128.8 (6)	C37—P3—C49—C54	−164.6 (2)
C6—C1—C2—C3	0.0	C43—P3—C49—C54	88.5 (3)
P1—C1—C2—C3	176.8 (12)	Ag1'—P3—C49—C54	−55.0 (4)
C1—C2—C3—C4	0.0	Ag1—P3—C49—C54	−38.7 (3)
C2—C3—C4—C5	0.0	C37—P3—C49—C50	14.2 (3)
C3—C4—C5—C6	0.0	C43—P3—C49—C50	−92.7 (3)
C4—C5—C6—C1	0.0	Ag1'—P3—C49—C50	123.8 (4)
C2—C1—C6—C5	0.0	Ag1—P3—C49—C50	140.2 (2)
P1—C1—C6—C5	−176.7 (12)	C54—C49—C50—C51	0.6 (4)
C1—P1—C1'—C2'	42 (16)	P3—C49—C50—C51	−178.2 (2)
C7—P1—C1'—C2'	87.7 (8)	C49—C50—C51—C52	0.1 (5)
C13—P1—C1'—C2'	−167.3 (7)	C50—C51—C52—C53	−0.8 (5)
Ag1—P1—C1'—C2'	−41.0 (9)	C51—C52—C53—C54	0.8 (5)
C1—P1—C1'—C6'	−129 (17)	C52—C53—C54—C49	−0.1 (5)
C7—P1—C1'—C6'	−83.8 (7)	C50—C49—C54—C53	−0.6 (5)
C13—P1—C1'—C6'	21.2 (8)	P3—C49—C54—C53	178.3 (2)
Ag1—P1—C1'—C6'	147.5 (5)	C61—P4—C55—C60	6.4 (3)
C6'—C1'—C2'—C3'	0.0	C67—P4—C55—C60	−98.9 (3)
P1—C1'—C2'—C3'	−171.8 (11)	Ag1'—P4—C55—C60	148.0 (4)
C1'—C2'—C3'—C4'	0.0	Ag1—P4—C55—C60	130.7 (3)
C2'—C3'—C4'—C5'	0.0	C61—P4—C55—C56	−172.8 (2)
C3'—C4'—C5'—C6'	0.0	C67—P4—C55—C56	81.9 (3)
C4'—C5'—C6'—C1'	0.0	Ag1'—P4—C55—C56	−31.3 (4)
C2'—C1'—C6'—C5'	0.0	Ag1—P4—C55—C56	−48.6 (3)
P1—C1'—C6'—C5'	171.1 (13)	C60—C55—C56—C57	0.7 (5)
C1—P1—C7—C12	21.1 (7)	P4—C55—C56—C57	−180.0 (2)
C13—P1—C7—C12	−87.2 (3)	C55—C56—C57—C58	0.3 (5)
C1'—P1—C7—C12	18.1 (7)	C56—C57—C58—C59	−0.6 (6)
Ag1—P1—C7—C12	143.9 (2)	C57—C58—C59—C60	−0.1 (6)
C1—P1—C7—C8	−162.3 (7)	C56—C55—C60—C59	−1.5 (5)
C13—P1—C7—C8	89.4 (3)	P4—C55—C60—C59	179.3 (3)
C1'—P1—C7—C8	−165.2 (7)	C58—C59—C60—C55	1.2 (6)
Ag1—P1—C7—C8	−39.5 (3)	C67—P4—C61—C62	−163.8 (3)
C12—C7—C8—C9	0.7 (5)	C55—P4—C61—C62	90.3 (3)
P1—C7—C8—C9	−176.1 (2)	Ag1'—P4—C61—C62	−52.5 (4)
C7—C8—C9—C10	0.3 (5)	Ag1—P4—C61—C62	−37.6 (3)
C8—C9—C10—C11	−1.3 (5)	C67—P4—C61—C66	15.5 (3)
C9—C10—C11—C12	1.2 (5)	C55—P4—C61—C66	−90.4 (3)
C10—C11—C12—C7	−0.2 (5)	Ag1'—P4—C61—C66	126.8 (4)
C8—C7—C12—C11	−0.7 (5)	Ag1—P4—C61—C66	141.7 (3)
P1—C7—C12—C11	175.9 (3)	C66—C61—C62—C63	0.4 (6)
C1—P1—C13—C18	87.4 (6)	P4—C61—C62—C63	179.8 (3)
C7—P1—C13—C18	−168.0 (2)	C61—C62—C63—C64	−0.1 (6)
C1'—P1—C13—C18	85.3 (6)	C62—C63—C64—C65	0.0 (6)
Ag1—P1—C13—C18	−38.6 (3)	C63—C64—C65—C66	−0.1 (6)
C1—P1—C13—C14	−95.8 (6)	C64—C65—C66—C61	0.4 (6)
C7—P1—C13—C14	8.8 (3)	C62—C61—C66—C65	−0.6 (5)
C1'—P1—C13—C14	−97.8 (6)	P4—C61—C66—C65	−179.9 (3)
Ag1—P1—C13—C14	138.3 (2)	C61—P4—C67—C72	96.1 (3)
C18—C13—C14—C15	0.7 (4)	C55—P4—C67—C72	−157.7 (2)
P1—C13—C14—C15	−176.2 (2)	Ag1'—P4—C67—C72	−29.2 (3)
C13—C14—C15—C16	−0.1 (5)	Ag1—P4—C67—C72	−26.8 (3)
C14—C15—C16—C17	−0.2 (5)	C61—P4—C67—C68	−78.6 (3)
C15—C16—C17—C18	−0.1 (5)	C55—P4—C67—C68	27.5 (3)
C14—C13—C18—C17	−1.0 (5)	Ag1'—P4—C67—C68	156.0 (3)
P1—C13—C18—C17	176.0 (3)	Ag1—P4—C67—C68	158.4 (2)
C16—C17—C18—C13	0.8 (5)	C72—C67—C68—C69	−1.9 (5)
C31—P2—C19—C20	−156.3 (6)	P4—C67—C68—C69	172.8 (3)
C25—P2—C19—C20	97.4 (6)	C67—C68—C69—C70	0.8 (5)
C19'—P2—C19—C20	−81 (76)	C68—C69—C70—C71	0.9 (6)
Ag1'—P2—C19—C20	−14.4 (10)	C69—C70—C71—C72	−1.5 (6)
Ag1—P2—C19—C20	−30.6 (8)	C70—C71—C72—C67	0.3 (5)
C31—P2—C19—C24	21.1 (8)	C68—C67—C72—C71	1.4 (5)
C25—P2—C19—C24	−85.2 (7)	P4—C67—C72—C71	−173.6 (3)
C19'—P2—C19—C24	97 (76)	O1—C73—C74—F2	85.3 (7)
Ag1'—P2—C19—C24	163.0 (6)	O2—C73—C74—F2	−93.6 (9)
Ag1—P2—C19—C24	146.8 (6)	O1—C73—C74—F1	−153.1 (6)
C24—C19—C20—C21	0.0	O2—C73—C74—F1	28.0 (10)
P2—C19—C20—C21	177.5 (11)	O1—C73—C74—F3	−33.1 (8)
C19—C20—C21—C22	0.0	O2—C73—C74—F3	148.0 (8)
C20—C21—C22—C23	0.0	O2'—C73'—C74'—F2'	31.8 (12)
C21—C22—C23—C24	0.0	O1'—C73'—C74'—F2'	−147.3 (12)
C22—C23—C24—C19	0.0	O2'—C73'—C74'—F3'	−86.3 (12)
C20—C19—C24—C23	0.0	O1'—C73'—C74'—F3'	94.5 (13)
P2—C19—C24—C23	−177.4 (11)	O2'—C73'—C74'—F1'	154.2 (11)
C31—P2—C19'—C20'	−172.4 (5)	O1'—C73'—C74'—F1'	−25.0 (15)
C25—P2—C19'—C20'	81.1 (6)		

### Hydrogen-bond geometry (Å, º)

**Table:**

*D*—H···*A*	*D*—H	H···*A*	*D*···*A*	*D*—H···*A*
O3—H3···O1	0.84	2.41	2.728 (8)	104
O3′—H3′···O1′	0.84	2.03	2.72 (2)	138
